# President’s Address, McLennan Co. Medical Society*Delivered at Waco, December, 1909.

**Published:** 1911-08

**Authors:** M. B. Saunders


For Texas Medical Journal.

### President's Address, McLennan Co. Medical Society.*

BY M. B. SAUNDERS, M. D.

[Having recently moved from Waco to San Antonio and associating myself with the county society here I find its members have many ideas in common with the gentlemen I left behind; and as on some of these points I have radically different views, I hope I will be pardoned at this late date offering my retiring address to the press for publication.]

Gentlemen: It gives me pleasure to see you here tonight, and I wish to thank you for the interest you have shown and for the part you have taken in making our meeting most pleasant and instructive.

I had hoped this year to have instilled into the members of this society the importance of the study of practical therapeutics and show how we are losing ground by neglecting this, the most important branch of our calling. The very object of our association is to establish a rallying point for the dissemination of therapeutical knowledge; and, as we grasp hands and look into each other's eyes we should feel inspired with an intense desire to do good and to communicate it.

We are living in an extraordinary period in the history of medicine. You and I have witnessed, many of us have helped in the two great epoch making events which will stand out in the future as the most prominent in the history of medicine, the organization

*Delivered at Waco, December, 1909.

of the American medical profession, and the State's regulation of the practice act. On the other hand, our pharmaceutical brothers have not been inactive, for the first time the United States Pharmacopoeia (8th revision) has been adopted by the United States government in its entirety as a standard. And more far reaching than this the passage of the pure food and drug act of June 30, 1906.

Gentlemen, the Pharmacopoeia is a medical book of the first rank, created by medical men for the establishment of a standard. Entering a little into its history: In 1817, Dr. Laymon Spalding submitted a project to the Medical Society of New York for the formation of a pharmacopoeia. The society approved the movement and issued circulars requesting the co-operation of the several State medical societies, the medical schools and colleges and voluntary efforts of physicians and surgeons to assist in the undertaking. Every ten years this book has been carefully revised by physicians. Not wishing to burden you with details we will drop down to May 7, 1890. We find in the city of Washington 175 delegates present, representing the medical department of the United States army, medical department of the United States navy, medical department of the marine hospital service, and fifteen medical societies, twenty-three medical colleges, twenty-four pharmaceutical associations, and twenty-three colleges of pharmacy, for the purpose of revising and bringing up to a standard the United States Pharmacopoeia. So we see as I have said it is indeed a medical volume of the highest merit.

Yet I find in my association with physicians that they know very little about this book and almost universally their criticism is, "I find in the Pharmacopoeia many things which I never use, and find absent many things that I do use." The reason for this is plain. The great mass of us have wandered far afield for many years, and have prescribed all kinds of medicines, made by all kinds of manufacturers, taking up one specialty after another, often because we were seized by an irresistible desire to use the latest thing out. We try the many samples furnished us by the manufacturers, and the result has been destructive to therapeutics founded upon true scientific facts.

Talk with your druggist and you will find that his shelves are loaded with unremunerative stock left upon his hands by this habit, because they have been compelled to buy specialties which were being used by the doctors, and after trying them for a short while, we are induced to take up something different, possibly no better, but we wanted to try it, and so it has come about the scientific therapeutics is nearly dead, and confusion and doubt has taken the place of the knowledge and wisdom acquired by careful systematic study of the effect of medicines, the composition of which was known, so that if we are doing nothing else there being lost a great amount of time and effort, browsing among fields that leads to this uncertainty.

These facts may not be new to you, and new facts are not needed; the old facts are sufficient for this generation, but what is needed is a wise guidance in the future. Let us be practical—we are dealing with problems of existence. And when I started on my career as your president I tried to start this movement but found myself "like the voice of one crying in the wilderness." There is an army of men now abroad in our land meeting physicians, urging and demonstrating to them that if they will retrace their steps, study the Pharmacopoeia and National Formulary, using in their practice only official remedies, then they can place therapeutics on a firm foundation, because they will be prescribing remedies whose composition is known and with this goes the assurance that the official preparations comply now with the standard, for we now have laws which compel the druggist to supply official remedies. It is a campaign of constructive work—the slogan now is, "Get together," and why should not the profession of medicine and pharmacy join hands? Why could not this society have a meeting arranged with our druggists, ask them to get out their percolators, filter stands, mortars and apparatus and make for us the official preparations, and we would use them, and if we wanted some additions made, what better chance would the pharmacist ask than a chance to co-operate, and what matters the cost if both doctor and druggist devote some time to such co-operation? Increase respect for each other, greater confidence in prescribing by the doctor and vastly better relations between the two, and what about the patient? No longer will they feel that the druggist is only a merchant, and the doctor no better than the quack, for has not the doctor prescribed and the druggist dispensed a proprietary, the taste of which is perfectly familiar to the patient, who has been feeding on the same stuff for weeks with no relief, and now the doctor's fee for professional advice is added to his misery, so he hopelessly turns his face to the wall.

Gentlemen, if I can only get you to realize the truthfulness of what I have said—get you to thinking—it is no wonder that we are taking refuge in nihilism and like the lotus eater's dream that all is folly, that rest and quiet and calm are the only human fruition.

The surgeon studies his books and knows well his technique and looks down upon the family practitioner as a good fellow but not a scientific man; small fees are good enough for him, but better pay must be the surgeon's. Thus the lack of therapeutic knowledge makes us feel like strangled snakes about the cradle of Hercules. Yet I tell you the family practitioner has infinitely more difficulties to surmount, and it is this lack of confidence in ourselves that causes us to bundle up our dear ones and seek aid in those with whom we are less familiar. Mistakes, yes, they all make mistakes; the one as foolish as the other; only the surgeon's work is more spectacular. I thoroughly agree with Sir Frederick Treves that "the actual manipulative part of surgery required no great skill, and many an artisan shows infinitely more adaptness in his daily work. A wood engraver would probably find as little difficulty in baring the carotid artery as a stone carver would find in performing an osteotomy." So why this pinnacle that we place the surgeon? And right here I wish to go on record that the surgeon should be the family physician's hand maid, to be called in by the family physician, for it is he who has to diagnose whether or not he is needed, and dismissed by the family physician. The fee to be collected by the physician who in turn settles with the surgeon. We thus see the family physician calls in an assistant to help him in bringing back the patient to health. And the family physician remaining supreme in the eyes of his client. It has been suggested that the family physician so embarrasses the surgical attendant that he can not be at his best. To those thus affected I should say it is proof positive that they realize their own weakness and had rather not the family physician be cognizant of that fact. To me I prefer him, and insist on his continual attendance if possible. * * * Well do I remember an incident in my early career. I was stopping in a cross roads town when a typical pine woods man came in hunting his doctor. He was not to be found, and being young in my calling I offered my services. I shall never forget his reply, and the longer I practice medicine and study human nature the more I see the wisdom of his remark: "You may be the best doctor in all Texas, but I do not know you, so I will wait for my family physician."

Gentlemen, if we would all practice with this spirit the family physician would not be losing ground, the so-called specialist would not strip him of the confidence of his clients, a better feeling of patient to his physician, a better feeling of physician to surgeon, and if "toted fair" with a better feeling all around for each other.

"Why talk of independence, there is no such word,
For we depend upon each other for everything that life is worth."

Gentlemen, we are not all physicians who have license to practice, and here I am only speaking to the gentlemen we respect—the trained therapeutists I think will understand me.

So, gentlemen, in parting I hope what I have said may cause us to think and start a third epoch in organized medicine. The study and application of therapeutics, thus restoring to us that confidence in ourselves and in our patients, and adding to the dignity of our calling. Now, before leaving, if I have during my term of office offended any one by word or deed I ask your forebearance.

